# An explanatory sequential mixed method study of nursing students’ self-efficacy in caring for older adults in Ghana

**DOI:** 10.1371/journal.pone.0334404

**Published:** 2025-12-02

**Authors:** Diana Abudu-Birresborn, Martine Puts, Lynn McCleary, Charlene H. Chu, Vida Yakong, Lisa Cranley

**Affiliations:** 1 Department of Nursing, Faculty of Applied Health Sciences, Brock University, St. Catharine’s, Ontario, Canada; 2 Lawrence Bloomberg Faculty of Nursing, University of Toronto, Toronto, Ontario, Canada; 3 KITE-Toronto Rehabilitation Institute, University Health Network, Toronto, Ontario, Canada; 4 School of Allied Health Sciences, University for Development Studies, Tamale, Northern Region, Ghana; Yonsei University Medical Center: Yonsei University Health System, KOREA, REPUBLIC OF

## Abstract

**Background:**

There is considerable evidence indicating that nursing students demonstrate inadequate knowledge and negative attitudes toward working with older adults. This suggests nursing student’s unpreparedness to provide care for the expanding older adult population. Feelings of unpreparedness can negatively impact their motivation and confidence. However, limited evidence exists about how nursing students’ knowledge and attitudes influence their self-efficacy in caring for older adults. Knowing this can help to identify gaps and opportunities to facilitate nursing students’ confidence in caring for older adults in acute care settings.

**Aim:**

To examine nursing students’ knowledge, attitudes, and self-efficacy and how these variables impact nursing student self-efficacy in caring for older adults in acute care settings in Ghana.

**Methods:**

We employed explanatory sequential mixed method approach. In Phase I, we used a cross-sectional design and collected quantitative data about students’ knowledge, attitudes, and self-efficacy. Data were collected from 170 second and third-year nursing students between December 2019--March 2020. We analyzed the data using descriptive and multiple-variable linear regression. Survey results informed the selection of students for Phase II based on their scores. In Phase II, 17 nursing students were purposively selected for semi-structured interviews between November and December 2020. Interviews were transcribed and analyzed using thematic analysis. Both results were integrated and presented.

**Results:**

Students’ mean age was 21 years (SD = 3.73). Just over half were female (54%). The majority had lived with/were currently living with older adults (83.0%). Many had low knowledge scores (71%) and a majority had positive attitudes (91%) and high self-efficacy scores (97%). Nursing students’ ages and attitudes were significantly positively associated with their self-efficacy. There was no significant association between students’ gerontology knowledge and self-efficacy. Qualitative findings showed that low knowledge scores were due to limited attention to gerontology education in the curriculum and heavy course load. Sociocultural norms in caring for older adults influenced students’ positive attitudes. This facilitated students’ interactions with older adults and increased their confidence. Higher self-efficacy scores were associated with the impact of the general nursing program, students’ perceived familiarity with the needs of older adults and routine procedural knowledge. Younger students perceived that their age and competencies were questioned by older adults, impacting their self-efficacy. Both datasets converged at integration.

**Conclusion:**

It is imperative to enhance students’ knowledge and leverage their self-efficacy to advance gerontological nursing education and practice in Ghana.

## Introduction

Researchers have explored nursing students’ knowledge, attitudes, and motivations for working with persons 60 years and above to understand and identify ways to improve students’ interest in caring for older adults [1,2]. However, students continue to rank gerontology as one of the lowest career choices of interest for practice or specialty [1,3–7]. Difficulty communicating with older adult patients, heavy workload, and inadequate knowledge about caring for older adults are among the main reasons nursing students do not prefer gerontological nursing as a career path [3,5,8–10]. Due to the growing ageing population and age-related changes in physical, functional, and cognitive health needs, older adults are increasingly seeking healthcare services [11–13]. Because of the lack of long-term care or senior nursing home facilities in Ghana, older adults seek healthcare services in acute care settings as the general population [12,14]. General nurses and midwives provide nursing care in these acute care facilities in Ghana with no specialized gerontology training and are expected to provide competent and quality care to older adult patients. The acute care setting has patients with complex health problems across the lifespan. The concerns and needs of ‘at risk’ patients such as older adults may not be optimally addressed [15].

Nursing education provides nursing students with cognitive and psychomotor skills development, enhancing their theoretical and experiential knowledge acquisition [16]. This is expected to help develop nursing students’ competencies and self-confidence for future practice. Findings of a scoping review [6] about nursing students’ preparedness to care for older adults in lower- and middle-income countries (LMICs) showed that nursing students may perceive themselves as inadequately prepared. Feeling unprepared to care for older adults can negatively impact nursing students’ motivation and self-belief or confidence to care for older adults [17].

Bandura’s self-efficacy theory guided this study. The theory suggests that a person’s perception of their self-efficacy- of how competent they are likely to be in a given task, influences their choices, commitment, and efficiency in their interactions with others and the task [18]. Self-efficacy is task specific, and persons with high sense of self-efficacy will pursue and continue with a task irrespective of how challenging that task is. They persist in being effective in their interactions and become successful in the task [18,19]. Persons with low self-efficacy will avoid a task they were not able to complete in the past and may not pursue it further [19]. Nursing students with low self-efficacy in caring for older adults will not commit to working with them. They may avoid or delay interventions resulting in serious negative outcomes [20,21]. Bandura recommended four ways to enhance and improve self-efficacy: guided mastery, vicarious learning, social persuasion, and self-regulation. These self-efficacy factors are congruent to teaching and learning gerontological nursing and may enhance students’ self-belief and interest in caring for older adults.

There is abundant literature on nursing students’ self-efficacy in areas such as cultural competency [22,23], learning motivations and academic performance [24,25], the impact of simulation practice [16,26–28], the impact of preceptorship and clinical instructor [29–31] and communication and urinary catheterization [32,33]. However, there is limited evidence about nursing students perceived self-efficacy in caring for older adults. Specifically, the association between students’ knowledge and attitudes on their self-efficacy to care for older adults is unknown in LMICs. This paper reports findings from a mixed-method study undertaken as doctoral research with the following questions: What are nursing students’ perceptions of their gerontology content knowledge levels in acute care settings in Ghana? What are students’ attitudes toward older adults in Ghana? And what are students’ understanding of their self-efficacy in caring for older adults? [34]. Understanding nursing students’ perceptions about their gerontology content knowledge, attitudes, and self-efficacy may help to recognize and identify opportunities, strategies, and gaps in gerontological nursing education and practice that can inform the development of interventions to advance gerontological nursing practice in Ghana.

## Materials and methods

### Study design

An explanatory sequential mixed method design was employed [35].

**Fig 1:**
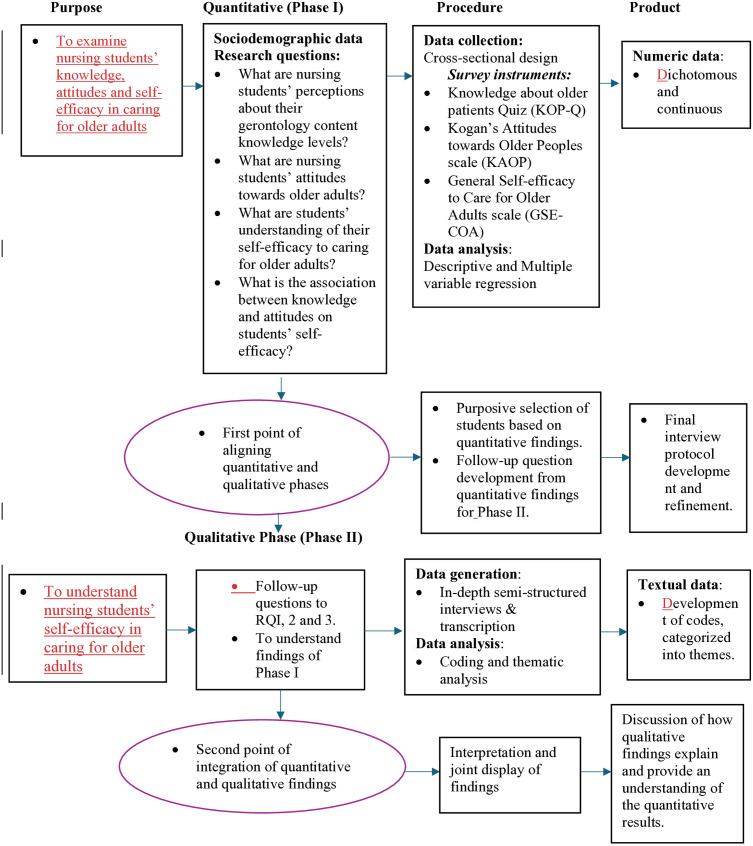
Explanatory Sequential Mixed-Method Approach.

Quantitative survey data were first collected and analyzed, followed by the collection and analysis of qualitative interview data. The data were integrated and interpreted [35,36]. In the quantitative phase (Phase I), a cross-sectional design was used for data collection to examine nursing students’ content knowledge, attitudes toward older adults and their self-efficacy in caring for them. The findings informed the refinement of the interview guide and selection of participants for the qualitative data generation (Phase II). Phase II explored how nursing students’ content knowledge and attitudes influenced their understanding of their self-efficacy in caring for older adults. The findings were jointly tabulated and presented to explain the quantitative results and identify convergence or divergence for a broader understanding of nursing students’ self-efficacy in caring for older adults in Ghana. Fig 1 presents the study design.

### Study sampling

#### Quantitative (Phase I).

A two-stage sampling method was employed. The first stage involved the site recruitment of participating institutions. The two broad northern and southern geographic sectors of Ghana were used as a guide for site recruitment. The inclusion criteria for site recruitment were:

1) A public Nurses’ and Midwives’ Training College (NMTC) offering the three-year Diploma Nursing Certificate Program. A single national curriculum guides the 3-year program by Ghana’s regulatory body of nursing education and practice [37].
2) Nurses’ and Midwives’ Training College affiliated with a Teaching Hospital. The five Teaching Hospitals in Ghana serve as referral points from regional hospitals and are affiliated with universities and colleges for training health professionals.

A convenience sampling of two public Nurses’ and Midwives’ Training Colleges (NMTCs) accessible to the PI, based on cost and feasibility, were recruited [38].

The second stage involved sampling participants. Participants were recruited from December 3rd, 2019 – March 11^th^, 2020. Second- and third-year nursing students were sampled as they had more clinical experience to provide their perspectives on their self-efficacy in caring for older adults than first-year nursing students. Information sessions about the study’s aim and purposes were conducted in the second and third-year classrooms. Students asked questions about their rights as participants, including confidentiality and whether they had the right to drop out at any time. Participation was voluntary. Those who volunteered to participate were given a package in an envelope containing coded questionnaires, two copies of the information letter/informed consent, and a thank you card. Alphanumeric codes that contained non-personal descriptors and study site locations were assigned to questionnaires and consent forms. This allowed the principal investigator (DAB) to align students’ responses in Phase I to Phase II should they agree to participate in the second phase. At the end of the consent form was an invitation to participate in phase II.

#### Sample size estimation (Phase I).

The target sample size was calculated using the rule of thumb estimation for sample size in multiple linear regression analysis and the eight predictors and covariates from our larger study [39,40]. Thus, N = 50 + 8k, [40], where N = sample size, k = the number of predictors and covariates. Using a reported 70% and above response rate among nursing students [1,4,41–43], the sample size was calculated and rounded to 170 second and third-year nursing students.

### Study instruments

**Socio-demographic Data**: The selection of sociodemographic variables was based on previous research [1,41] that reported that age, gender, years of school, living with/having lived with an older adult, religion, and where they come from (region) (southern/northern sector), influence nursing students’ motivations and interest in working with older adults. All the data except for age and religion were dichotomous and were collected to describe the population and determine their impact on nursing students’ self-efficacy in caring for older adults.

**Nursing Students’ Self-Efficacy:** The General Self-Efficacy to Care for Older Adults (GSE-COA) scale modified from the General Self-efficacy (GSE) for the specific task of caring for older adults was used [39,44]. The 26-item scale is rated on a five-point Likert scale, with responses ranging from 1 (not very like me), 2 (not like me), 3 (not sure), 4 (like me), and 5 (very like me). Scores are summed for a total score range between 26–130. Using the middle response as a split point, 78 was used as the cut-off point [45]. Participants who had scores higher than 78 suggest high self-efficacy. Scores below 78 indicate low self-efficacy. The overall Cronbach Alpha coefficient for the modified GSE-COA scale was 0.85 and is considered high [46,47].

**Knowledge about Older Patients Quiz** (KOP-Q): The KOP-Q is a unidimensional scale comprising 30 true or false items [48,49]. Due to the lack of a validated survey tool to measure nursing students’ content knowledge in caring for older adults in our study context, we adapted the KOP-Q scale [50]. The modified scale comprises 15 unidimensional items. A correct response scored +1, and an incorrect response scored zero. The scores are summed for a total score range between 0–15. Scores of 7.5 and above were considered adequate, and scores lower than 7.5 were inadequate using Bloom’s cut-off point [45]. The KR-20 for the original scale was 0.70 [48]. In this study, the Kuder-Richardson-20 (KR20) reliability was 0.30, which is considered low [46].

**Kogan’s Attitude Towards Old People Scale (KAOP**): The KAOP [51] originally consisted of 32- items rated on a 5-point Likert scale, ranging from 1 strongly disagree to 5 strongly agree. We evaluated and adapted the scale for our study context [50]. The adapted KAOP scale consists of 22 items on a 5-point Likert Scale. Half of the statements are negative, and the other half are positive regarding attitudes toward older individuals [52]. Negative statements were reverse coded for one total positive score ranging between 22 and 110. The maximum score for a neutral response of 66 was determined as the cut-off point [45]. Higher scores above 66 indicated positive attitudes, and lower than 66 indicated negative attitudes. The Cronbach alpha coefficient of 0.65 and is considered moderately satisfactory [46].

#### Qualitative (Phase II).

Participants who completed the invitation to participate in Phase II were sampled purposefully from November 5^h,^ 2020 – December 19^th,^ 2020. Using mean scores as cut-off points, nursing students who scored above and below the mean for knowledge, attitudes, and self-efficacy were contacted via phone and recruited for Phase II [36,45]. Nursing students were sampled until data saturation, where no new information or patterns were noted from subsequent participants during analysis [53,54]. Seventeen nursing students were purposively selected to participate in Phase II.

### Ethical considerations

The study was conducted following ethics approval from the University of Toronto Health Sciences Research Ethics Board (ERB) (#37347) and the Ghana Health Service Ethics Review Board (GH-ERB) (#GHS-ERC-001/06/19). Permission was sought and approved by the Regional Health Directors (RHD) and Principals of the NMTIs to recruit nursing students. Written informed consent was obtained from nursing students who participated in the study.

### Inclusivity in global research

Additional information regarding the ethical, cultural, and scientific considerations specific to inclusivity in global research is included in the Supporting Information (S1 Checklist).

### Data collection procedures

#### Quantitative (Phase I).

Completed questionnaires were dropped in a sealed box left at the College reception and picked up by the principal investigator daily after school hours. Some students handed over completed questions to the PI on site. Questionnaires were examined for completeness, and data were coded and entered into SPSS IBM Version 26.

#### Qualitative (Phase II).

Students who completed the invitation form for Phase II and provided their; emails/phone numbers for follow-up interviews also dropped them in the sealed box. The completed invitation forms were identified, sorted, and matched with the student’s knowledge, attitudes, and self-efficacy scores using the alphanumeric codes on the questionnaires. Students were contacted, and interviews were conducted with those who agreed to participate. The PI conducted the interviews on the phone due to Covid-19 pandemic restrictions. Students were asked to locate a quiet, non-interruptive environment for the phone interviews. Interviews were audio recorded and lasted 45–60 minutes. Alphanumeric codes and pseudonyms were used to protect participants’ identities and maintain confidentiality.

### Data analysis

#### Quantitative (Phase I).

Quantitative data analysis was conducted using SPSS IBM version 26. Missing values were examined using descriptive statistics and missing values analysis in SPSS to determine the proportion, type, and pattern of missing data. The proportion of missing data was less than 5% of all the cases and excluded from the analysis [55,56]. Descriptive and multiple linear regression analysis was conducted. Descriptive statistics examined nursing students’ knowledge, attitudes, and self-efficacy in caring for older adults. The following hypothesis guided regression analysis.

H0: Knowledge about and attitudes toward older adults is not associated with nursing students’ self-efficacy to care for older adults.

H1: Knowledge about and attitudes toward older adults are associated with nursing students’ self-efficacy to care for older adults.

### Multiple regression assumptions testing

**Fig 2:**
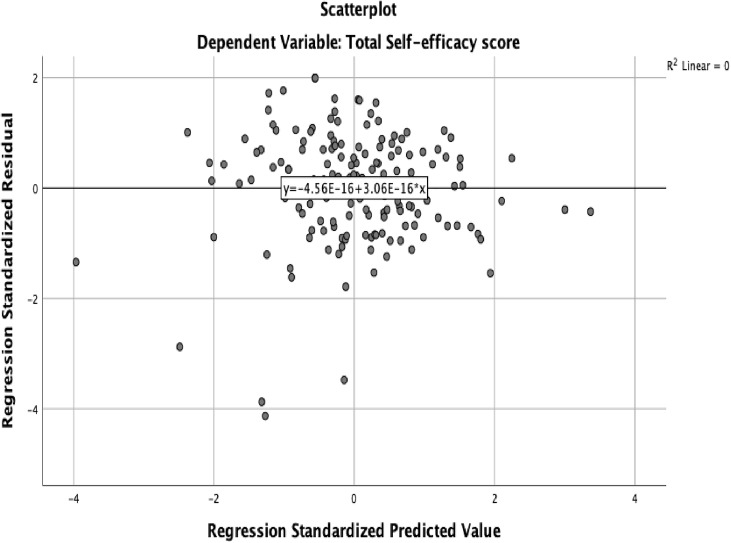
Scatter plot for linearity.

The assumptions of a multiple variable linear regression analysis (linearity, normality, independence of error, homoscedasticity, and multicollinearity) were evaluated to ensure that the data were appropriate. The linearity assumption stipulates that a linear relationship should exist between the dependent variable and independent variables [40,57]. We used scatter plots and standardized residuals and predictors to examine linearity. The scatter plot showed a linear relationship between self-efficacy, knowledge, attitudes and the socio-demographic data. The linearity line showed that standardized residual and standardized predictors were around zero suggesting a linear relationship. The linearity assumption was satisfied, see Fig 2.

**Fig 3:**
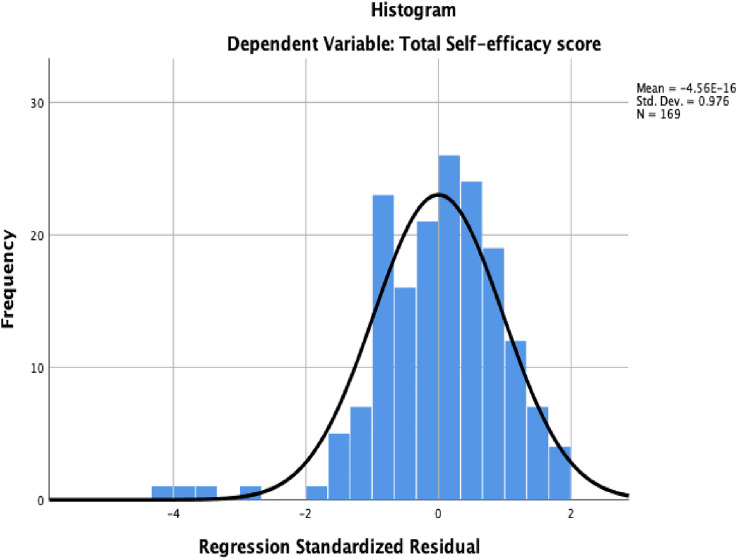
Histogram of standardized residual.

**Fig 4:**
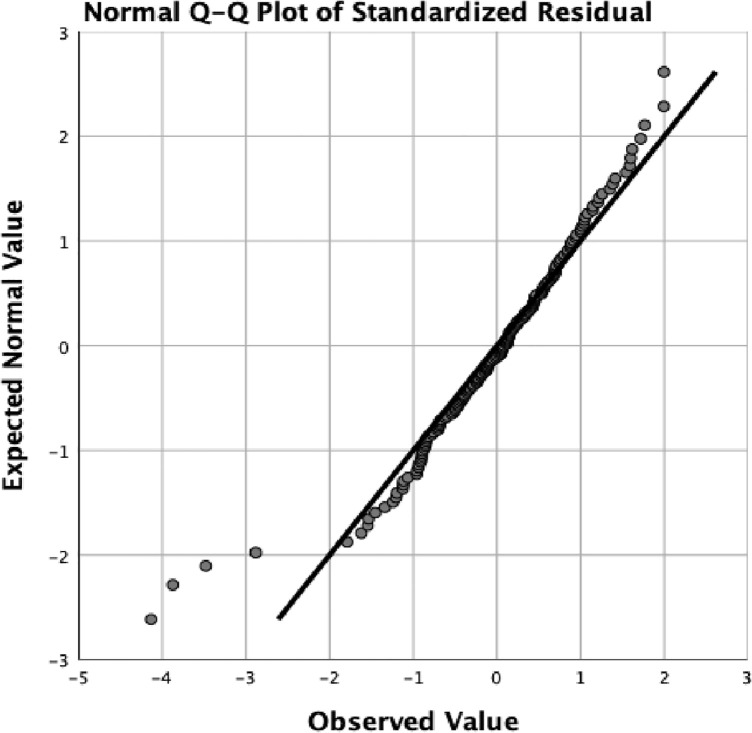
Q-Q Plot of standardized residual.

Normality assumption indicate that the residuals of the dependent (outcome) variable should be normally distributed [40,57]. The assumption was assessed using histogram and Q-Q plot, see Figs 3 and 4. Visual inspection of the histogram suggested a distribution of a normal curve with a slight negative tail. Normality of the residuals were further examined with the visual inspection of the Q-Q plot. As Q-Q plot are sensitive to deviations in the tails of distribution, they are effective in identifying potential outliers if they are present in the data [57].

The Q-Q Plot shows the data points cluster around the horizontal line with the tail slightly pulled to the negative. The presence of outliers appears to violate the normality assumption [40]. Examination of the data for entry errors showed that the extreme values are legitimate data points. Using IBM SPSS explore, the distribution of the variables was examined to identify the outliers. Five cases (S003, S080, S021, S018 and S053) were identified to have extreme values. Outliers were further examined using Mahalanobis distance in regression analysis [40]. The *P*-value of the right tail of chi-square was calculated using 1-CDF.CHISQ(MAH_1, 8) to create a new probability variable. With the use of *p* < .001 criterion for Mahanalobis distances, cases S097, S015, S086, S021, S061 and S1117 had probability values of (.0000) respectively. Removal of these cases did not have any influence on the distribution of the normality curve; and were maintained. The extreme values which were due to the age variations of the participants present a true representation of the population distribution.

**Fig 5:**
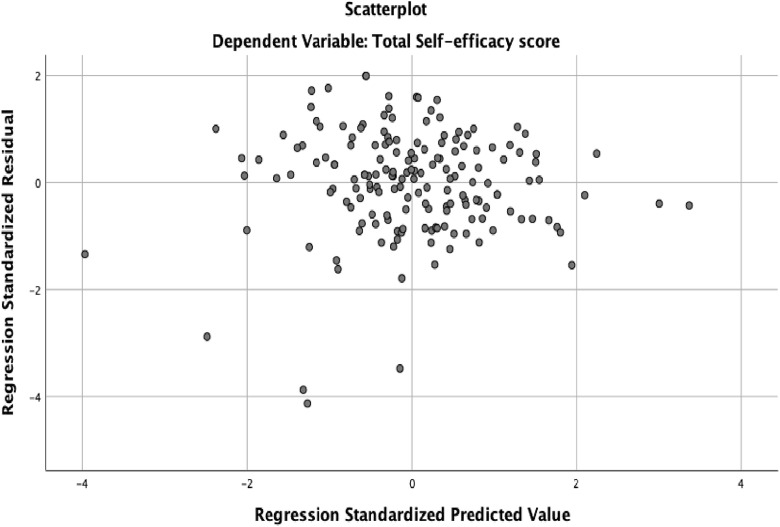
Scatter plot of standardized residuals for independence of errors.

Independence of error was assessed using visual examination of the standardized residuals plots and the Durbin-Watsons statistic. The residuals appeared randomly scattered and did not present a funnel shape pattern. The Durbin-Watsons score ranges from 0–4, with values around 2 indicating independence of errors. In this study, the Durbin-Watson score was close to 2 supporting the assumption of independence of errors, see Table 1. Thus, the assumption of independence was satisfied, see Fig 5.

**Table 1:** Model Summary^b^.

Model	R	R Square	Adjusted R Square	Std. Error of the Estimate	Durbin-Watson
1	.469a	0.22	0.181	12.928	1.863

a Predictors: (Constant), Have you lived with or currently living with an older adult?

What is your gender, Age in years, Total knowledge Scores, Total Attitude Scores,

Which sector in Ghana is your institution located, what year are you in school? What is your religion.

b Dependent Variable: Total Self-efficacy score.

Homogeneity of error variance (homoscedasticity) assumption indicate that error variance should be constant across all levels of predictor variables [40]. This was examined using scatter plot of the standardized residuals and standardized predictors. The scatter plot showed that the variance is randomly scattered around zero suggesting homogeneity of variance. See Fig 5. The test of homogeneity of error variance was satisfied.

Multicollinearity assumption requires that two or more of the predictor variables are not correlated with each other [40]. This ensures that the individual contribution of each independent variable to the variance explained by the dependent variable can be accurately interpreted. In this study, multicollinearity was evaluated using the Variance Inflation Factor (VIF) values. The VIFs ranged from 1.07 to 1.51 which is below the accepted threshold of 10, indicating the absence of multicollinearity, see Table 2.

**Table 2:** Collinearity of independent variables.

Independent variables	Collinearity Statistics	
	Tolerance	VIF
Total knowledge Scores	0.927	1.078
Total Attitude Scores	0.953	1.049
Age in years	0.952	1.051
What is your gender	0.813	1.23
What is your religion	0.661	1.513
Which sector in Ghana is your institution located What year are you in school?	0.782	1.279
Have you lived with or currently living with an older adult?	0.841	1.189
a Dependent Variable: Total Self-efficacy score	0.837	1.195

Results of the assessment of the assumptions of linearity, normality, independence of error, homoscedasticity and multicollinearity were all met. The normality assumption was met with some few extreme values on age pulling the tail of the curve to the negative. The extreme ages of some of the participants are a representation of the population and was maintained for analysis.

#### Qualitative (Phase II).

Interviews were transcribed and organized by the PI using NVivo 12. Analysis was done using thematic analysis and guided by self-efficacy theory, research questions, and existing literature [58,59]. We employed thematic analysis (TA) and used deductive and inductive approaches to coding [59,60]. Using the deductive approach, coding and recording were from general to specific terms. For example, in examining students’ knowledge, attitudes, and self-efficacy, *general terms like ‘knowledge’ ‘attitudes,’ ‘confidence,’ and ‘self-efficacy’* were identified as codes when discussed by participants. Specific terms or phrases like *‘do not know’ ‘I am prepared,’ and ‘I am confident’* were determined as codes. During inductive coding, coding and recoding were from specific to general using terminology and terms from the participants’ [61]. Participants’ terms, phrases, or terminology were implied based on the study context and helped in the generation of codes. For example, in this phrase… “*you know at home, the grown-ups will tell you don’t talk back to an aged person, you should bow and greet them. So, when you apply all this in the ward, sometimes it helps too”.* This was coded as ‘attitude’ as the student draws on sociocultural teachings to facilitate interactions and positive behaviour toward older adult patients.

In addition, deliberate analytic decisions were made throughout the analysis process by reading, rereading, and developing analytic questions during coding and constructing shared meanings [59,62]. To illustrate, analytic questions like; *how do nursing students’ experiences about working with older adults shape their understanding of their self-efficacy in caring for older adults?* asked during coding helped to identify similar concepts and the meanings and assumptions participants ascribed to their responses. In answering the above analytic question, the PI recognized that students adopt various self-regulatory mechanisms, including viewing older adults as their parents/grandparents which helps them control their emotions and cope with challenging experiences when working with older adults.

Through this self-regulatory approach, students who successfully complete a task for a perceived ‘difficult’ or ‘uncooperative’ older adult viewed themselves as confident which shaped their perceived self-efficacy in caring for older adults. Thus similar responses and assumptions were grouped into categories and themes were generated [63]. Employing analytic questions highlighted how the participants’ cultural and social backgrounds influenced their views about their self-efficacy in caring for older adults in Ghana. The analytic questions also highlighted students’ positions in their responses as learners and/or caregivers of older adult relatives at home which enhanced the data interpretation process [61].

### Rigour, positionality, and reflexivity

A reflexive journal of the decisions and experiences after interviews was maintained to enhance credibility. The reflexive journal allowed the PI to be conscious of assumptions and prejudices that may impact data collection. The systematic documentation of the study design, implementation activities, and processes enhanced dependability and confirmability [64,65]. Transferability was achieved through the reflexivity and positionality of the PI and the research process. The PI’s identity as a female, trained Ghanaian nurse and doctoral student was altered between insider and outsider positions during interviews [66]. The PI’s theoretical positioning, including motives, personal history, disciplinary affiliation, and a preceptor, was acknowledged which enhanced the study’s credibility [60,67]. Additionally, providing sufficient information about the context of the study and participants enhanced transferability by allowing readers of the study to decide whether the findings were relatable and transferable to their contexts.

## Results

### Quantitative (Phase I)

Nursing students’ knowledge about older adults, attitudes toward older adults, and self-efficacy to care for older adults were examined with frequencies and descriptive statistics. There was a 99% response rate. The results are presented in Table 3.

**Table 3:** Socio-demographic, Knowledge, Attitudes, and Self-Efficacy Scores.

Characteristic	Frequency (F)	Percentage (%)	Mean (SD)
**Age in years**			21.3 (3.73)
**Gender**			
Male	78	46.2	
Female	91	53.8	
Choose not to answer			
**Religion**			
Muslim	61	36.1	
Christian	108	63.9	
Traditional religion			
Other			
**Sector of institution**			
Northern	84	49.7	
Southern	83	49.1	
Choose not to answer			
**Year of school**			
Second year	83	49.1	
Third year	83	49.1	
**Lived with/ living with an older adult**			
Yes	140	82.8	
No	27	16.0	
Choose not to answer			
**Knowledge (KOP-Q)**			6.54 (2.01)
Inadequate knowledge	120	71.0	
Adequate	49	29.0	
**Attitudes (KAOP)**			77.3 (8.61)
Negative attitudes	16	9.5	
Positive attitudes	153	90.5	
**Self-efficacy (GSE-COA**)			107.96 (14.30)
Low self-efficacy	5	3.0	
High self-efficacy	164	97.0	
**Total (N = 169)**			

F- Frequency, N- Sample size, SD-Standard deviation.

**KOP-Q**- Knowledge of Older Patient Quiz.

**KAOP**- Kogan’s Attitudes Towards Old Peoples Scale.

**GSE-COA**- General Self-Efficacy to Care for Older Adults Scale.

From Table 3, the majority of students n = 120 (71%), had a mean score of 6.5 (SD = 2.01), suggesting inadequate knowledge about the care of older adults (Table 1). Regarding students’ attitudes, most students, n = 153 (90.5%), had a mean score of 77.1 (SD = 8.61), indicating positive attitudes. Most students, n = 164 (97%), also had a mean of 107 (SD = 14.29), suggesting a high self-efficacy.

The association between knowledge about and attitudes toward older adults on nursing students’ self-efficacy in caring for older adults in acute care settings was examined using multiple variable regression analysis. The findings are shown in Table 4.

**Table 4:** Association between knowledge, attitudes, and Self-efficacy (outcome variable) N (169) Nursing students.

	B	Std. Error	Sig.	95% confidence intervals	
				Lower Bound	Upper Bound
Variables		13.208	0.00	13.226	65.395
Knowledge	−0.306	0.516	0.56	−1.326	0.714
Attitude	0.749	0.119	0.00	0.515	0.984
Age	0.559	0.274	0.04	0.018	1.101
Gender	−1.76	2.212	0.43	−6.129	2.608
Religion	1.164	2.547	0.65	−3.866	6.195
North/South Ghana location	0.973	1.182	0.41	−1.362	3.307
Year in school	−1.348	0.979	0.17	−3.282	0.586
Lived with/ currently living with an older adult?	1.846	1.178	0.12	−0.479	4.172
Constant	39.31	13.208	0.00	13.226	65.395
*N = 169* P* < 0.05					

The self-efficacy model was significantly associated with nursing students’ self-efficacy in caring for older adults and accounted for 18% of the variance, see Table 1. Knowledge about older adults was not significantly associated with self-efficacy (*b* = −0.31, [95% CI −1.3, 0.7], p > .05). However, nursing students’ attitudes and age were significantly associated with their self-efficacy in caring for older adults respectively (*b* = 0.75, [95% CI 0.5,1.0], p < 0.05), (*b* = 0.56, [95% CI 0.0, 1.0], p < .05), see Table 4.

### Qualitative findings (Phase II)

To understand and explain the findings of Phase I, nursing students’ gerontology knowledge, attitudes toward and self-efficacy in caring for older adults were explored. Three main themes; perceived content knowledge, perceived attitudes, and perceived self-efficacy and subthemes were generated and are discussed in Table 5. below.

**Table 5:** Qualitative Findings.

Theme	Subtheme	Quote
**1: Perceived Content Knowledge about Caring for Older Adults** Nursing students acknowledged their knowledge deficits in gerontological nursing and recognized the need to acquire theoretical knowledge citing the nursing training institutions as responsible for preparing them adequately. They underscored that the nursing training institutions pay little attention to gerontological nursing and suggested the need to build the faculty’s capacity to educate them [nursing students] appropriately. Students argued that attention is more on courses, including medical-surgical nursing, obstetrics and gynecology, pediatrics, and public health with comprehensive contents and clinical placement components. In further acknowledging their knowledge deficits, students often discussed their perceived knowledge about caring for older adults in three areas: gerontological content knowledge, routine procedural knowledge, and a common-sense approach and perceived familiarity in caring for older adults.	**1: Nursing Students’ Gerontological Content Knowledge** Students attributed their inadequate content knowledge to a heavy course load, limited attention to gerontology courses, inadequate time to complete the gerontological nursing curriculum, inadequate gerontology content and limited gerontological practical skills development. Heavy course load and limited time to complete the gerontological nursing course were frequently highlighted in the interviews. Nursing students believed that the general nursing education curriculum was heavy and focused more on clinical hours, impacting the teaching, and learning of gerontology. They noted that time constraints to complete the current gerontology content impact their gerontology learning experience resulting in difficult experiences when working with older adults. **2: Routine Procedural Knowledge and Common-sense Approach** In contrast to content knowledge, nursing students believed that they had adequate clinical procedural nursing knowledge. They considered gerontological nursing to require procedural skills and discretion to provide nursing care services, including bed bathing and feeding. As a result, nursing students believed that the necessary skills they acquired from their skills lab training in providing nursing care services were adequate to care for older adults. These nursing care services are limited to routine procedural knowledge. Nursing students discussed that because of their knowledge of routine procedures, they are perceived as competent by staff nurses, who sometimes call them to come to work if needed. In Ghana, assigning students to patients during clinical placements is often left to the discretion of the unit manager. The nurses recognize and compliment students’ efforts at work and sometimes call students as support staff. Therefore, nursing students transfer their perceived routine procedural knowledge of caring for the general adult patient to provide care for older adults. However, nursing students also recognized that applying procedural knowledge from the skills lab and transferring that to provide care for an older adult is sometimes challenging. Procedural knowledge emphasizes steps in carrying out nursing tasks and activities for the general adult patient population. Hence, nursing students draw on a common-sense approach or reasoning and modify the procedural knowledge to care for older adults. They indicated that although they knew the steps from practicing with mannequins in the skills labs, they drew on reasoning to complete tasks for an older adult patient; they suggested the need for gerontological skills development in the skills lab. **3: Perceived Familiarity with the Needs of Older Adults** Nursing students framed their knowledge about caring for older adults based on their perceived familiarity with the needs of older adults. They viewed caring for older adults as not ‘difficult’ and of less value because of their exposure to older adults at home. It is not uncommon to have older adults living with their children and other family members in Ghana. Students indicated that they understood the needs of older adults because of their interactions with them and have lived with or supported older adult relatives at home. They described these needs as providing privacy, compassion, and respect, including addressing older adults by their titles and family roles; Alhaji/grandfather and providing them with attention.	*“I don’t have enough knowledge and skills to take care of the older patients, I know I don’t have enough theoretical knowledge, but I need more knowledge and skills on taking care of an older patient, and I think the school has to pay attention to them to teach us.”(Participant 2, 2nd yr. KOP-Q score 5/15)* *“…….. I lack some knowledge of caring for aged people. And because of the course load, our curriculum (general nursing curriculum) is voluminous, so, the teachers don’t get enough time to explain certain things and complete the curriculum. So, in dealing with older adults normally, we face some difficulties.” (Participant 7, 3rd yr. KOP-Q score 6/15).* *“I think what we are supposed to know, we have been taught, bed bathing, feeding,* *communicating and what again…. hmmm, I think we have been taught what we need to care for them (older adults).” (Participant 6, 3rd yr, KOP-Q score 7/15).* *“Sometimes you will be in the house, they (staff nurses) will call you to come and help them in the hospital and the aged too, are part of the patients.” (Participant 9, 3rd yr, KOP-Q score 7/15).* *“We learn the various advanced nursing procedures using mannequins for all patients. So, when we get to the ward, we transfer what we have learnt to care for them (older* *adults)”. (Participant 6, 3rd yr KOP-Q score 7/15).* *“So, when we go to the wards, we can transfer our knowledge, and sometimes to* *excuse me for saying you have to add your common sense to care for the older patients.” (Participant 2, 2nd yr, KOP-Q score 5/15).* *“The dummies [mannequins], it helps. One to know what to do. But when you meet the real aged person, you see that no, you should do this and this and this, as in, you have to add somethings…. should I say reasoning.” (Participant 13, 3rd yr KOP-Q score 8/15).* *“Learning to care for older adults is not that difficult because you’ve been, we’ve been living with some of them at home, and you know what they want and don’t want. So, I consider their privacy when working with them. Also, apart from privacy, I will say I make sure they’re comfortable with me, as much as possible, so that I can carry out my procedure well.” (Participant 3, 2nd yr. KOP-Q score 6/15).* *“They [older adults] want to be addressed by their titles. So sometimes you see the person when you even call them, like in my local dialect when you call him ‘mba’, which translates as my father- but the person is Alhaji (gone for pilgrimage to Mecca), they’ll prefer you call them Alhaji than mba (My father). So sometimes they also prefer the titles to the usual grandfather, and the moment you start by bringing those titles, you see they have more concentration than before. Then you can relate with them.” (Participant 8, 3rd yr. KOP-Q score 6/15).*
**2: Perceived Attitudes Toward Older Adults** Nursing students’ attitudes toward older adults were shaped mainly by their clinical perspectives of older adults who require nursing care services. Students’ attitudes toward older adults were also influenced by societal expectations of how to behave towards older adults, and they drew on these sociocultural norms to interact and relate with older adults, which they presented as positive attitudes. However, students’ experiences interacting with and caring for older adult patients also impacted their attitudes toward them. These experiences were mainly perceived as negative, resulting in ageist attitudes toward older adults.	**1: Nursing Students’ Views of Older Adults** Nursing students had varied views about older adults. Some students constructed their views about older adults as being i: dependent - ‘older adults act like a child or a kid,’ weak and fragile, ii: talkative – ‘complain about everything, nosy, authoritative, iii: isolated- lonely and bored, angry for no reason, and forgetful- indecisive and unable to understand things easily. These views about older adults influenced nursing students’ behaviors and attitudes toward older adults. For example, nursing students stated that they needed to have patience to care for older adults. Therefore, they adopted actions or behaviors to control their emotions in anticipation of perceived inappropriate behavior by an older adult patient like shouting and being uncooperative with them. This anticipated behavior by older adults required nursing students to regulate their emotions to provide care for older adults. Some nursing students view older adults as “children” because they [older adults] are perceived as uncooperative, dependent and do not understand things easily, which requires students to be patient to work with them. These perceived views and attitudes are illustrated in the following excerpts. **2: Societal Expectations (Social Desirability)** Sociocultural norms and expectations shape the values and attitudes of nursing students as they interact with and care for older adults, both at home and in health facilities. Guided by these expectations and their professional training, students exhibited culturally expected attitudes toward caring for older adults. They acknowledged the sociocultural teaching of being respectful to every person and more so with older adults, because older adults were perceived as old as their parents or grandparents and must be respected and supported. **3: Ageism** Discrimination, neglect, and abuse of older adults are common forms of prejudice or stereotypical behaviour against older adults, which are expressions of ageism. Nursing students described that delayed or missed care occurs for reasons such as state of illness, frailty, and lack of time. Nursing students’ attitude toward older adults was framed based on how long it would take to complete a nursing task for an older adult. Students described that caring for older adults was time-consuming. They had to do everything for older adults because they [older adults] are frail. This perception of older adults as frail results in decisions to postpone care for older adults due to the time it would take to complete tasks and not on the personal needs of the older adult patient. Postponing the care needs of older adults due to the time it takes to complete tasks was a typical ageist behaviour portrayed by nursing students towards older adult patients. Students preferred caring for younger adult patients because young patients facilitated the nursing care process and were not dependent on nursing students to do everything for them.	*“The general [societal view] thing is older adults are weak, older adults cannot do this, especially, when they are critically ill cannot do things for themselves, they are fragile.” I don’t know if it is because they’re old. You can see that they’re lonely, want people to talk to, and want to talk with people outside their family.”* *(Participant 16, 2nd yr. KAOP score 76/110).* *“You have to exercise patience dealing with them because when you are expecting them [older adults] to behave, they will do as if they are children, or they don’t want to get well and go home.” (Participant 9 3rd yr. KAOP score 78/110).* *“So, it’s like hmmm when they become aged… they still behave like...a child or something. Their understanding is quite different and far.” (Participant 11, 3rd yr. KAOP score 77/110).* *“I think for me; respect is very important. Having respect for older adults, even though it cuts across all stages of life, whether a child or adult, you must respect, but with older adults, you must respect them more and give them the maximum respect they need. Because that is what our parents teach us, that person (older adult) can be your grandfather or grandmother and even your mother or father.” (Participant 4, 2nd yr. KAOP score 80/110).* *“So, it’s like caring for older adults… it takes time to carry out other tasks. Yeah, unlike maybe a young boy like me, like they come one, two, three, you are done with the procedure, and then he (young patient) will even help you do some things, and then you are done, and you go. But with older people, they may not help, and it’ll take a very long time. So sometimes, when you see older people on the ward, you have to finish with other things before you come to them.” (Participant 16, 2nd yr KAOP score 76/110).*
**3: Perceived Self-efficacy to Care for Older Adults** In this study, self-efficacy was used synonymously with confidence. Nursing students believed they had high self-efficacy in caring for older adults. They framed their perceived confidence to care for older adults based on the totality of the general nursing education program and their perceived familiarity with older adults. Nursing students explained that they were trained to be general nurses, implying their ability to care for all patient groups, including older adults, as a duty and a responsibility. In discussing their perceived confidence, students explained that they draw on content knowledge from other courses, including medicine, advanced nursing, and therapeutic communication, to interact and care for older adults. They also framed their confidence based on their procedural knowledge from their experiences in the skills laboratory and clinical placements. Students recognized that their skills lab and clinical placements were general learning experiences with limited focus on gerontological nursing clinical placement. Furthermore, nursing students’ age impacted their perceived self-efficacy in caring for older adults. Younger nursing students felt that older adult patients often questioned their competence, impacting their self-efficacy.	**1: Totality of the General Nursing Education Program** Some students considered courses such as gerontology, traditional medicine and home care nursing, basic and advanced nursing, therapeutic communication, and medicine as beneficial to their overall preparation in caring for older adults. They believed that these courses and their clinical placement experience made them confident to care for all patients, including older adults. Students drew on their skills lab training focused on basic and advanced nursing skills and competency development for their perceived confidence in procedural knowledge and exposure to clinical environments. Nursing students perceived caring for older adults as not different from the general adult population and requiring basic and advanced nursing competencies. **2: Nursing Students’ Age and Self-efficacy in Care for Older Adults** Nursing students described their self-efficacy in caring for older adults concerning their age. Nursing students noted that older adult patients perceived them as young and compared them to their adult children. Students explained that when older adults viewed them as young, it created a discomfort barrier, between them and the older adults. This made students want to avoid them [older adults] when possible. They explained that being young and providing invasive nursing procedures, including catheterization and bed bathing, makes the older adult patients uncomfortable, impacting their confidence level in providing care for older adults.	*“I know that is my duty and my responsibility as a nurse. So, there is no need to resist or avoid taking care of older adults. It is my responsibility and my duty to take care of older adults too. Once you are in the hospital or they are in my hands, they are everything; I will take good care of them, so I will do all the best that I can do.”* *(Participant 9, 3rd yr. GSE-CAO score 121/150).* *“So as for my confidence level, that one is very high. I don’t know why I should be afraid or doubt myself when taking care of what we call an older patient. Because that patient could be your mother or your father, so when you are taking care of your father, why should you fear.”* *(Participant 11, 3rd yr. GSE-CAO score 123/150).* *“I am confident with my nursing skills. I think when you follow the steps in the nurse’s manual, you should not have a problem caring for older adults. We have been taught how to do bed baths, feeding, and all…so you apply your knowledge and the skills like you do with* *other patients when you meet the elderly too.“(Participant 15, 3rd yr. GSE-CAO score 124/150*). *“Some of them also consider like because they have children like us, so it’s not… for you to see their nakedness…they are uncomfortable…Ahaa, that’s why most of them find it very difficult to let you do like catheterization and sometimes even bed bathing. Because you are like their children’s age, you ask yourself if when you are a nurse is this how it will be or because you are a student. And you don’t want to go there [older adult bedside] again.” (Participant 1, 2nd yr. 20yrs).* *“The older people, too, always see us as their children like we’re younger, so we* *must respect them. But sometimes, the way they will look at you because you are young when it is like that you too you just let them be…like bed bathing for instance…me I will just call their relatives to do it.“(Participant 12, 2nd yr.19yrs).*

### Data integration

Data integration occurred at two points. First, during data collection with the purposive sampling of students based on their scores in Phase I, to identify issues or factors to explain the quantitative results. The second integration occurred after data analysis of both quantitative and qualitative phases. The findings were compared to identify convergence or divergence. No point of divergence was noted in the integration of quantitative and qualitative data. Convergence occurred as qualitative findings identified issues and factors that supported and explained the quantitative results, providing a broader understanding of nursing students’ self-efficacy in caring for older adults in Ghana, see Table 6.

**Table 6:** Joint display of explanatory sequential mixed method results.

Research question	Quantitative results	Qualitative findings	Integration of results	Convergence
What are nursing students’ perceptions of their gerontology content knowledge levels in acute care settings?	Of the 169 nursing students, 120 (71%) scored below the cut-off point of 7.5 for knowledge about caring for older adults, suggesting inadequate content knowledge.	Nursing students’ insufficient content knowledge was attributed to heavy course load in the nursing program, inadequate time to complete the gerontology course, limited attention to gerontology education, inadequate gerontology content and practical skills development.	Nursing students acknowledged they had inadequate knowledge to care for older adults and identified the factors that impacted their content knowledge.	Yes.
What are nursing students’ attitudes toward older adults in acute care settings?	The majority, 153 (90.5%) of nursing students, scored above the cut-off point for attitudes, suggesting positive attitudes towards older adults.	Nursing students’ attitudes towards older adults were shaped based on their views of their understanding about the needs of older adults. Nursing students’ views about older adults influenced the way they interacted with older adults.	Nursing students indicated that they understood the needs of older adults, including showing respect, reciprocity, and compassion. Nursing students suggested that these perceived needs were socially expected and facilitated their interactions with older adults positively.	Yes.
What are nursing students’ understanding of their perceived self-efficacy to care for older adults?	The majority, 164 (97%), of nursing students had higher scores above the cut-off point of 78. This suggested a perceived high self-efficacy to care for older adults.	Nursing students suggested that their perceived confidence to care for older adults was due to the totality of the general nursing education program, familiarity with older adults at home and, their perceived competence in routine procedural nursing care.	Students with a high sense of self-efficacy were not afraid. They perceived older adults as their relatives, did not doubt their ability to care for older adults, had experience/exposure to older adults at home, and were comfortable working with them. Furthermore, they were willing to overcome any challenge working with older adults and perceived caring for them as their professional duty and responsibility.	Yes.
What is the association between nursing students’ knowledge about, attitudes towards, on self-efficacy to care for older adults?	Knowledge about caring for older adults was not significant to self-efficacy (B = −0.31, p > .05, [95% CI −1.3, 0.7]), implying no association between knowledge and self-efficacy.	Nursing students perceived routine procedural knowledge and perceived familiarity with the needs of older adults shaped nursing students perceived self-efficacy to care for older adults even though the majority had content knowledge scores below the cut-off point.	Nursing students indicated that their ability to care for older adults was based on their familiarity with the needs of older adults from home and their competencies in routine nursing procedural skills. Nursing students suggested that insufficient content knowledge about older adults did not impact their ability to care for older adults. Therefore, the finding provides an understanding for the lack of statistically significant association between content knowledge and self-efficacy.	Yes.
	Nursing students’ attitudes toward older adults contributed significantly to self-efficacy (*b* = 0.75, [95% CI 0.5, 1.0], p < 0.05), suggesting there was an association between attitudes and self-efficacy to care for older adults.	Nursing students’ experiences of working with older adults influenced their attitudes towards older adults. Students exhibited culturally acceptable attitudes towards caring for older adults, which were reflected in their attitudes as needing to be patient and care for older adults as their family members or relatives.	Nursing students’ attitudes were shaped by sociocultural norms and the perception of older adults as their relatives. By perceiving older adults as their relatives, students were expected to reciprocate the care they would have provided to their older adult parents or grandparents. Also, demonstrating these sociocultural norms facilitated their interactions and enhanced their confidence in providing care for older adults.	Yes.
	Nursing students’ age was a significant (*b* = 0.56, [95% CI 0.0, 1.0], p < .05). contributor to self-efficacy, implying an association between higher age and higher levels of self-efficacy to care for older adults. The mean age was 21years.	Nursing students indicated that being young and providing invasive nursing procedures, including catheterization and bed bathing, makes the older adult patient uncomfortable, impacting their confidence level when caring for older adults.	Nursing students indicated that being young and providing invasive nursing procedures, including bed bathing, makes older adults uncomfortable. They indicated that older adults questioned their competencies and associated their competencies with their age. Judging their competencies by their age impacted nursing students’ confidence in caring for older adults.	Yes.

## Discussion

We examined the association between nursing students’ knowledge and attitudes on their self-efficacy in caring for older adults in acute care settings in Ghana. Specifically, we examined students’ perceptions of their content knowledge and attitudes and explored their understanding of their self-efficacy in caring for older adults. Results showed that nursing students’ gerontology knowledge was insufficient and not statistically significant to self-efficacy. Students demonstrated positive attitudes toward older adults, however, they exhibited ageist perceptions and demonstrated compassionate ageism. Students’ attitudes and ages were significantly positively associated with self-efficacy. Students exhibited a high sense of self-efficacy, attributed to the broad skills and confidence they acquired in the general nursing program and routine procedural clinical knowledge.

The lack of statistically significant association between nursing students’ knowledge and self-efficacy was congruent with previous studies among nursing students in areas like managing pediatric pain, evidence-based practice, using patient simulation and caring for older adults in a nursing home [27,47,68,69]. The performance of the KOP-Q in our study population may explain our study outcome. The KOP-Q had a low KR20 reliability which might have impacted our finding. The low reliability was not surprising. This is because the reduction of the items from the original scale and the unrelated multiple concepts often measured in knowledge scales likely accounted for the low KR-20 reliability [46]. Other factors including guessing, which might have impacted the reliability of the KOP-Q are discussed in detail elsewhere [50]. However, the face and content validity of the KOP-Q were achieved through expert and participant validity assessment [70]. The expert of four panel in gerontological nursing education and practice in Canada and Ghana, allowed for the identification and inclusion of the appropriate items for the development of the survey instrument [71]. The readability, interpretation and comprehension of the items were further enhanced through cognitive interviewing and pilot testing of the survey instrument [71,72]. Although the KOP-Q had a low KR-20 reliability, the findings are valid as they measured substantive acute care gerontology content. The finding of insufficient content knowledge levels is consistent with previous reviews and empirical quantitative findings in LMICs [6,7,10,46,50,73–76]. The insufficient knowledge levels were further corroborated in the qualitative results of this study. Future researchers in LMICs should consider refining the KOP-Q to improve its reliability in LMICs, including Africa. This is because there are limited alternative validated survey tools for measuring gerontology knowledge in acute care settings in LMICs. The Palmore’s Facts on Aging Quiz, has widely been used for measuring knowledge about older adults and could likely be an alternative to KOP-Q [77]. However, the Facts on Aging Quiz has been criticized for measuring perceptions and includes items not specific to acute care settings [49].

Prior scholars suggested that sociodemographic data, years in school, living with/having lived with an older adult, impacted students’ interest and willingness to care for older adults [1,41]. However, these predictors did not yield statistically significant association between students’ self-efficacy and their knowledge. Our qualitative findings revealed that other factors, including nursing students’ knowledge of routine clinical nursing care, age, students’ procedural knowledge of performing tasks like bed bathing, feeding and their familiarity with the needs of older adults shaped their perceived self-belief and knowledge about caring for older adults. Some researchers noted interests in working with older adults, is also impacted by the work environment and working experience [1,41,78]. Future researchers should consider other contextual variables influencing students’ self-efficacy in caring for older adults.

Previous studies corroborate our study’s finding of positive attitudes toward older adults [2,74]. Researchers argued that nursing students who lived with older adult relatives had more positive attitudes toward older adults [2,74]. Similarly in our study, many students had lived with or were living with older adults due to the extended family structure. Living with older adults plays a crucial role in the socialization of students and might have shaped students’ positive attitudes. Students also presented ageist perceptions (frail, weak, and acting like a child) and compassionate ageism. Coined by Binstock, compassion ageism occurs when younger people perceive older adults as needing help and more vulnerable than they are [79,80]. Comparable findings among nursing and other healthcare professional students are reported in earlier studies as determinants of ageism [81,82]. Research on ageism and its impact on the quality of nursing care for older adults has largely been conducted in high-income countries [81,83] with limited evidence in LMICs. Further research is required to understand ageism and how it influences the nursing care of older adults in LMICs, especially in sub-Saharan Africa.

Results of previous studies, congruent with our study findings showed a positive association between students’ age and attitudes toward older adults and their self-efficacy [47,84]. In this study, interviews revealed that sociocultural expectations facilitated students’ interactions with and made them comfortable in working with older adults which increased their self-efficacy. However, younger students noted that their competencies were questioned by older adults which impacted their self-efficacy negatively. Another underlying assumption regarding the discomfort of older adults receiving care from younger nursing students could be gender. In a recent systematic review, authors argued that gaps exist in the nurse-patient relationship with older adults because of gender barriers, which can result in discomfort [85]. Potential researchers could explore the association of gender and how it shapes nursing student’s self-efficacy in caring for older adults in acute care settings in LMICs including Ghana.

Students’ high self-efficacy scores were due to their perceived clinical routine knowledge, familiarity with the needs of older adults and the overall impact of the general nursing program. Students’ clinical routine knowledge was based on their perceived general nursing competencies, not specific to gerontological nursing competencies. Previous evidence similar to our study finding linked perceived high self-efficacy with perceived competency in carrying out a task [86–88]. Although insufficient in gerontology content knowledge, students’ clinical and personal knowledge from their experiences can facilitate interaction, comfort, and confidence in working with older adults [89,90]. However, this personal knowledge may also present worrying concerns about students’ views regarding the care for older adults as requiring minimal specialized skills and suggesting an ingrained societal view about the care of older adults. Given that self-efficacy is a task-specific construct, our findings were likely influenced by students’ situational and individual factors, which might have impacted their higher scores and simplistic views about caring for older adults without related content knowledge levels [27,68,69,91,92].

Additionally, the totality of the general nursing program enhanced students’ views about their self-efficacy. Students drew information from multiple courses, including, medical-surgical nursing, therapeutic communication, mental health, and basic and advanced nursing which enhanced their confidence in caring for older adults. However, information from these courses did not seem to influence students’ gerontology content knowledge scores, as their mean knowledge scores were low. This raises a crucial question about how these courses support students’ gerontological learning and their self-efficacy in caring for older adults. And highlights the crucial need for adequate integrated and standalone gerontology content in nursing education to increase students’ knowledge and confidence and advance gerontological nursing practice [89,93,94]. Furthermore, educators can consider simulation and scenario-based approaches as a potential strategy for teaching nursing students’ crucial skills and competencies for working with older adults to enhance their confidence [95,96].

### Limitation and strengths

The recruitment of two institutions was a limitation, which may impact the generalizability of the findings [97]. Therefore, this study should be replicated with more nursing training institutions for generalization across Ghana. The modified KOP-Q had a low KR20 reliability in our study population, which might have impacted the knowledge scores. However, it measured substantive gerontology content appropriate for our study. Qualitative findings provided a broader understanding of students’ gerontology knowledge gaps. Nonetheless, future research should test additional psychometric properties of the modified KOP-Q with a larger sample size to enhance its KR20 reliability. Another limitation was the impact of the COVID-19 pandemic on the qualitative data collection. Interviews were conducted via telephone, limiting body language and other nonverbal cues that could enhance data interpretation.

### Implications for education, research, practice, and policy

Our findings highlight broader policy and systemic barriers in LMICs and suggest a critical need for greater focus on gerontological nursing education and practice. The insufficient knowledge gaps suggest a foundational need in nursing students gerontological knowledge to bridge these gaps. It behooves on nursing education programs to intensify gerontological nursing education to adequately position nursing students in readiness for the increasing older adult population and epidemiological shift from acute infectious diseases to multiple long term chronic conditions such as multimorbidity in LMICs [98]. The findings draw attention to the need for educational interventions including curricula revisions to reflect current trends.

In addition, the aging policy in Ghana aims to provide incentives and attract healthcare professionals to gerontological practice, however, there is little evidence to suggest its implementation [99]. Government and policy makers should be committed to attracting healthcare professionals, through the development of policies and creating funding opportunities to support educational programs in gerontology and the provision of resources to facilitate research and clinical gerontological nursing practice. The findings also suggest a need to improve the nursing care of older adults in acute care settings, particularly in LMICs with limited specialized personnel or care facilities for older adults. Specialized units for older adults or Acute Care for Elderly (ACE) have shown to improve patient outcomes such as reduced functional decline and falls, increased home discharge, and improved patient and staff satisfaction [100,101]. Recognizing that the creation of specialized wards may be a long-term goal due to resource constraints, integrating interim, age-appropriate compassionate care for older adults in acute care settings is both necessary and possible.

In addition, acute care settings can benefit from specialized trained personnel in gerontological nursing to support and facilitate appropriate quality care for older adults and socialize nursing students adequately in caring for older adults. Furthermore, providing practicing nurses with the opportunities for continuous professional programs in caring for older adults can be a first step to improving geriatric care and the student gerontology experience. This can be achieved as a requirement for the renewal of licensed Personal Identification Numbers (PINs). Furthermore, ensuring the availability of resources can promote positive working environments and experiences for nurses and nursing students alike and lead to positive patient outcomes. Findings also emphasize a need for increased research to refine and develop validated survey tools appropriate for ageing research. To facilitate understanding of the challenges and needs of older adults and develop interventions to improve positive outcomes.

## Conclusion

Nursing students perceived high self-efficacy, their positive attitudes toward and insufficient content knowledge about caring for older adults, indicate a significant opportunity to advance gerontological nursing education and practice in Ghana. This study has identified sources of students’ insufficient knowledge and perceived self-efficacy that can guide policy changes and inform teaching and learning strategies to improve quality and positive patient outcomes in caring for older adults in acute care settings in Ghana.

## Supporting information

S1 Checklist Inclusivity in global research questionnaire. (DOCX)
